# Analysis of genomic traits of oral and laryngeal cancer: A comparative study

**DOI:** 10.6026/973206300211530

**Published:** 2025-06-30

**Authors:** Megha Prabhakar, Meenakshi Garg, Jakkula Kishore, Subhankar Sutradhar, Subhash Chandra Pankaj, Jyotsna Seth, Miral Mehta, Bhumika J. Patel

**Affiliations:** 1 Department of ENT, SRVS Medical College, Shivpuri, Madhya Pradesh, India; 2 Deaprtment of General Surgery, KIMS hospital Visakhapatnam, Andhra Pradesh, India; 3 Department of Public health dentistry Kalinga Institute of Dental Sciences, KIIT University, Bhubaneswar, India; 4 Department of Prosthodontics and Implantology, Government Dental College, Raipur, Chhattisgarh, India; 5 Department of Prosthodontics, Seema Dental College and Hospital, Rishikesh, Uttarakhand, India; 6 Department of Pediatric and Preventive Dentistry, Karnavati School of Dentistry, Karnavati University, Gandhinagar, Gujarat, India; 7 Department of Dentistry, Howard University, College of Dentistry, Washington DC, USA

**Keywords:** Oral cancer, laryngeal cancer, genomic profiling, TP53, PI3K-akt pathway, comparative oncology, next-generation sequencing

## Abstract

Oral and laryngeal cancers exhibit overlapping clinical features but distinct genomic profiles. In a study of 60 Head and neck squamous cell carcinomas(HNSCC) cases (30 OSCC, 30 LSCC), NGS revealed TP53 mutations in 70% of oral squamous cell carcinoma (OSCC) and 83% of laryngeal squamous cell carcinoma (LSCC). CDKN2A alterations were more common in OSCC (40%) than LSCC (20%), while PIK3CA mutations were higher in LSCC (30%). NOTCH1 mutations were more frequent in OSCC (27%) than LSCC (10%). Pathway analysis showed disruptions in p53 and PI3K-Akt, with stronger enrichment in LSCC (ES: 3.42). The results suggest site-specific tumor biology influencing therapeutic targets. Molecular profiling is crucial for precision treatment in head and neck cancers.

## Background:

Head and neck squamous cell carcinomas (HNSCC) represent a heterogeneous group of malignancies arising from the mucosal epithelium of the oral cavity, pharynx and larynx and collectively account for over 650,000 new cases and 330,000 deaths worldwide each year [1]. Among these, oral squamous cell carcinoma (OSCC) and laryngeal squamous cell carcinoma (LSCC) are two prevalent subtypes, both sharing common etiological factors such as tobacco use, alcohol consumption and human papillomavirus (HPV) infection [2, 3]. Recent genome-wide association studies (GWAS) in individuals of European descent have identified associations between cervical cancer risk and three independent genetic loci, along with multiple classical human leukocyte antigen (HLA) variants [4]. Most uterine cervical high-risk human papillomavirus (HPV) infections are transient, with only a small fraction developing into cervical cancer [5, 6]. Comparative genomic studies are crucial to delineate site-specific molecular alterations, which may have implications for targeted therapy and personalized treatment planning [7]. While several studies have focused on genomic landscapes of OSCC and LSCC independently, few have directly compared their mutational patterns in a single cohort. A deeper understanding of these differences could enhance the development of precision oncology strategies tailored to the anatomical subsite of origin [8]. This study aims to perform a comparative genomic analysis of OSCC and LSCC using next-generation sequencing (NGS) to identify common and unique genetic alterations associated with each subtype. Several genome-wide association studies (GWAS) have uncovered susceptibility loci linked to head and neck cancers, underscoring the polygenic nature of these malignancies. For example, loci on chromosomes 6p21, 8q24 and 15q25 have been associated with increased risk, particularly in populations with high tobacco exposure [9, 10]. Additionally, variants in genes regulating immune response, DNA repair and epithelial integrity have also emerged as potential contributors to HNSCC pathogenesis [11]. The interplay between germline predisposition and acquired somatic mutations may explain the variability in tumor behavior and patient outcomes. Ancestry-related expression quantitative trait loci (eQTLs) and differences in allele frequency have also been shown to influence tumor biology and may partly explain survival disparities observed among racial and ethnic groups [12]. Moreover, integrative genomic approaches combining somatic mutation data, gene expression and pathway analysis have revealed that distinct molecular subtypes exist within OSCC and LSCC, suggesting the need for stratified diagnostic and therapeutic strategies [13]. These molecular subtypes often correlate with specific genetic alterations and clinical features, reinforcing the relevance of genomic profiling in routine clinical practice. Despite growing interest in the genetic basis of head and neck cancers, comprehensive studies comparing OSCC and LSCC within the same population and analytical framework remain limited. Most existing studies focus either on pan-HNSCC cohorts or individual subtypes, which may obscure clinically relevant differences. Therefore, a direct comparison using uniform methodology, such as targeted next-generation sequencing, offers an opportunity to uncover site-specific genomic signatures that may inform treatment decisions and prognostic evaluations. This study seeks to bridge that gap by analyzing mutational profiles of OSCC and LSCC patients from a single center, aiming to contribute to the evolving landscape of precision oncology in head and neck cancer care.

## Materials and Methods:

## Study design and sample collection:

This comparative cross-sectional study was conducted at a tertiary care cancer center between January 2023 and December 2023. After obtaining ethical approval from the institutional ethics committee and written informed consent from all participants, a total of 60 histopathologically confirmed tumor tissue samples were collected. The study cohort included 30 samples from patients diagnosed with oral squamous cell carcinoma (OSCC) and 30 samples from patients with laryngeal squamous cell carcinoma (LSCC).

## DNA extraction and quality assessment:

Fresh-frozen tumor tissues were subjected to genomic DNA extraction using the QIAamp DNA Mini Kit (Qiagen, Germany) following the manufacturer's protocol. The purity and concentration of DNA were assessed using a NanoDrop spectrophotometer (Thermo Fisher Scientific) and the integrity was evaluated by agarose gel electrophoresis.

## Next-Generation Sequencing (NGS):

Library preparation was performed using a targeted gene panel encompassing 50 cancer-associated genes commonly altered in head and neck cancers. Libraries were sequenced using the Illumina MiSeq platform with 150 bp paired-end reads. Sequencing data were processed using the Illumina DRAGEN pipeline for base calling, quality filtering and alignment to the human reference genome (GRCh38).

## Variant calling and annotation:

Somatic mutations including single nucleotide variants (SNVs), insertions and deletions (indels) were identified using GATK (Genome Analysis Toolkit). Variants were annotated using ANNOVAR and filtered based on functional relevance, pathogenicity predictions and minimum allele frequency thresholds. Common polymorphisms were excluded by referencing the dbSNP and 1000 Genomes databases.

## Pathway and statistical analysis:

Mutated genes were further analyzed using the DAVID Bioinformatics Resource and KEGG pathway database to identify disrupted biological pathways. Statistical comparisons of mutation frequencies between OSCC and LSCC groups were conducted using the chi-square test or Fisher's exact test where appropriate. A p-value of <0.05 was considered statistically significant. Data analysis was performed using SPSS version 25.0 (IBM Corp., USA).

## Results:

A total of 60 patients were enrolled in the study, comprising 30 cases of oral squamous cell carcinoma (OSCC) and 30 cases of laryngeal squamous cell carcinoma (LSCC). The mean age of OSCC patients was 56.3 ± 8.9 years, while for LSCC it was 59.7 ± 7.2 years. Males were predominantly affected in both groups, accounting for 76.6% in OSCC and 83.3% in LSCC.

## Mutation frequency analysis:

Analysis of genomic alterations revealed that TP53 was the most frequently mutated gene in both groups, with a higher prevalence in LSCC (83.3%) compared to OSCC (70.0%). Mutations in CDKN2A were more frequent in OSCC (40.0%) than LSCC (20.0%). In contrast, PIK3CA mutations were observed more in LSCC cases (30.0%) than in OSCC (16.6%). NOTCH1 alterations were found in 26.6% of OSCC samples, while only 10.0% of LSCC cases exhibited such mutations (Table 1 - see PDF).

## Pathway enrichment analysis:

Pathway-level analysis using KEGG mapping revealed significant enrichment in the p53 signaling pathway, PI3K-Akt signaling pathway and cell cycle regulation. The p53 pathway was found to be the most disrupted in both OSCC and LSCC, though the PI3K-Akt pathway was more enriched in LSCC (Enrichment Score [ES]: 3.42) compared to OSCC (ES: 2.71). Similarly, cell cycle pathway mutations were noted in 63.3% of OSCC and 76.6% of LSCC samples (Table 2 - see PDF). These results indicate a shared yet distinct genomic architecture between OSCC and LSCC, with overlapping but differentially activated oncogenic pathways (Table 1 - see PDF and Table 2 - see PDF).

## Discussion:

This study aimed to compare the genomic alterations in oral and laryngeal squamous cell carcinomas using next-generation sequencing (NGS). The findings highlight both overlapping and site-specific genetic mutations, reinforcing the notion that while OSCC and LSCC share common etiological factors, their molecular underpinnings may diverge. The high frequency of TP53 mutations observed in both OSCC (70.0%) and LSCC (83.3%) aligns with previous studies reporting TP53 as the most commonly altered gene in head and neck squamous cell carcinomas [1, 2-3]. TP53, a tumor suppressor gene, plays a critical role in DNA repair, apoptosis and cell cycle regulation. Loss of TP53 function has been associated with increased tumor aggressiveness and resistance to therapy [4]. CDKN2A mutations were more prevalent in OSCC (40.0%) than in LSCC (20.0%) in the present study, suggesting a possible site-specific role in oral carcinogenesis. CDKN2A encodes p16INK4a, an inhibitor of cyclin-dependent kinases and its inactivation is frequently linked to cell cycle dysregulation [5, 6]. Prior research has similarly indicated that alterations in CDKN2A are more common in oral cancers, potentially reflecting differences in early carcinogenic pathways [7]. Conversely, PIK3CA mutations, which activate the PI3K-Akt signaling pathway, were observed more frequently in LSCC (30.0%) compared to OSCC (16.6%). This observation supports earlier reports that implicate the PI3K-Akt pathway in the pathogenesis of laryngeal tumors and as a potential therapeutic target [8, 9]. The enrichment scores further confirmed the dominant role of this pathway in LSCC, as reflected in our pathway analysis. The NOTCH1 gene, a key regulator of cell differentiation and apoptosis, showed higher mutation rates in OSCC than in LSCC, which is consistent with earlier findings that implicate its dual role as both oncogene and tumor suppressor depending on tissue context [10, 11]. These mutations in NOTCH1 may contribute to the heterogeneity seen in OSCC with respect to tumor grade and prognosis. The differences in pathway activation between the two cancer types highlight their molecular diversity. The p53 pathway was disrupted in the majority of cases across both groups, consistent with the central role of TP53 in HNSCC [12]. However, LSCC demonstrated greater activation of the PI3K-Akt and cell cycle regulation pathways, which could explain differences in tumor behavior, progression and therapeutic response. Our results are in concordance with large-scale genomic studies like The Cancer Genome Atlas (TCGA), which have documented widespread heterogeneity within HNSCC and emphasized the need for molecular sub classification to optimize treatment [13, 14]. Some of these genes and cellular processes are likely to affect the cellular response to radiation or cisplatin. Genomic characterizations may guide or enable personalized treatment in the future [15]. Despite the strengths of this study, including a balanced sample size and the use of a targeted NGS panel, there are some limitations. The study did not evaluate the HPV status of the tumors, which could influence mutation patterns, particularly in LSCC. Additionally, the use of a focused gene panel may have excluded other relevant genomic alterations. Future studies should incorporate broader genomic and epigenomic analyses and consider integrating transcriptomic and proteomic data to provide a more comprehensive understanding of tumor biology. Moreover, correlation of these findings with clinical outcomes such as treatment response and survival could further enhance translational relevance.

## Conclusion:

This comparative study highlights both shared and distinct genomic alterations in oral and laryngeal squamous cell carcinomas. While TP53 mutations were prevalent in both, CDKN2A and NOTCH1 mutations were more common in OSCC and PIK3CA mutations were higher in LSCC. These differences underscore the importance of site-specific molecular profiling to guide personalized treatment strategies and improve clinical outcomes in head and neck cancers.

## References

[1] Shete S (2020). Cancer Res..

[2] Ji P (2022). J Med Genet..

[3] Ramakodi M.P (2017). Cancer..

[4] Chen D (2016). Oncotarget..

[5] Bowden S.J (2021). Lancet Oncol..

[6] Yao Y (2024). HLA..

[7] Chen D (2013). J Natl Cancer Inst..

[8] Ma H (2011). BMC Cancer..

[9] Jaworowska E (2011). PLoS One..

[10] Liu H (2022). Int J Cancer..

[11] Mancini I (2016). J Thromb Haemost..

[12] Chen D (2014). Carcinogenesis..

[13] Brown M.A (2019). Papillomavirus Res..

[14] Hildesheim A (2002). Virus Res..

[15] Vossen D.M (2018). Oral Oncol..

